# Prognostic roles of metabolic reprogramming-associated genes in patients with hepatocellular carcinoma

**DOI:** 10.18632/aging.104122

**Published:** 2020-11-12

**Authors:** Lijuan Cui, Huan Xue, Zhitong Wen, Zhihong Lu, Yunfeng Liu, Yi Zhang

**Affiliations:** 1 Department of Pharmacology, School of Basic Medicine, Shanxi Medical University, Taiyuan 030001, China; 2 Department of Endocrinology, The First Affiliated Hospital of Shanxi Medical University, Taiyuan 030001, China

**Keywords:** metabolic reprogramming, prognostic, hepatocellular carcinoma, survival analysis, TCGA

## Abstract

Metabolic reprogramming for adaptation to the tumor microenvironment is recognized as a hallmark of cancer. Although many altered metabolic genes have been reported to be associated with tumor pathological processes, systematic analysis of metabolic genes implicated in hepatocellular carcinoma prognosis remains rare. The aim of this study was to identify key metabolic genes related to hepatocellular carcinoma, and to explore their clinical significance. We downloaded mRNA expression profiles and clinical hepatocellular carcinoma data from The Cancer Genome Atlas database to explore the prognostic roles of metabolic genes. Five prognosis-associated metabolic genes, including *POLA1, UCK2, ACYP1, ENTPD2,* and *TXNRD1,* were screened via univariate Cox regression analysis and a LASSO Cox regression model, which divided patients into high- and low-risk groups. Furthermore, gene set enrichment analysis revealed that significantly-enriched gene ontology terms and pathways involving high-risk patients were focused on regulation of nucleic and fatty acid metabolism. Taken together, our study identified five metabolic genes related to survival, which can be used to predict the prognosis of patients with hepatocellular carcinoma. These genes may play essential roles in metabolic microenvironment regulation, and represent potentially important candidate targets in metabolic therapy.

## INTRODUCTION

Hepatocellular carcinoma (HCC) is the most common primary liver cancer in adults, and is the leading cause of cancer-related mortality worldwide [1, 2]. It is well-known that HCC is a group of highly heterogeneous diseases, and the prognosis of individual patients varies greatly [2]. Clinically, tumor stage, histological grade, and molecular subtype are used to evaluate prognostic factors of patients with HCC. However, these clinicopathological features cannot accurately provide information to predict the prognosis, which may lead to inaccurate judgment regarding the prognosis and inappropriate treatment of patients [3, 4]. Therefore, there is an urgent need to identify new molecular markers to predict the prognosis of patients with HCC, which would be conducive in terms of accurate patient treatment.

Metabolic reprogramming is considered a new feature of cancer cells [5]. Catabolism and anabolism are key to cancer cells, which ensure their energy supply and biomass synthesis by reprogramming their microenvironment [6–8]. Numerous major metabolic pathways occur in the liver, including glycolysis, tricarboxylic acid cycle, oxidative phosphorylation, and amino acid catabolism [9]. Therefore, elucidating the relationships involving metabolism and HCC is essential for understanding the pathogenic mechanisms in tumorigenesis [10]. Previous studies have discussed the important role of metabolic disorders in cancer biology [11–13]. However, systemic metabolic reprogramming and its prognostic value in the progress of HCC need further study.

In this study, we used mRNA expression data of patients with HCC from The Cancer Genome Atlas (TCGA) database to develop a metabolic prognostic signature. These metabolism-related risk signatures can independently and effectively identify patients with HCC at a high-risk of unfavorable outcomes. In addition, performing gene set enrichment analysis (GSEA) determined the most relevant biological processes and metabolic pathways involved in the pathological process of HCC establishment. These results may provide new ideas for studying the prognosis of HCC, and for individualized metabolic treatment.

## RESULTS

### TCGA HCC dataset profile

We downloaded the data of 371 HCC cases from TCGA database, including 50 normal datasets and 374 tumor datasets. The tumor datasets were then randomly divided into a training set (n = 249) and a testing set (n = 125). After removal of cases with survival time < 30 days, or incomplete clinical information, a total of 221 cases with identified clinical characteristics were enrolled in this study. Clinical information concerning the patients in the training and testing sets is presented in Table 1. There were no statistical differences regarding clinical data between training and testing sets (*P-*value > 0.05).

**Table 1:** Clinical characteristics of patients.

**Variables**	**Training set (n=148)**	**Testing set (n=73)**	**Entire set (n=221)**	***P*-value**
**Age, n (%)**				0.052
< 65 yrs	95 (64.19)	53 (72.60)	148 (66.97)	
≥ 65 yrs	53 (35.81)	20 (27.40)	73 (33.03)	
**Gender, n (%)**				0.709
Female	45 (30.41)	24 (32.88)	69 (31.22)	
Male	103 (69.59)	49 (67.12)	152 (68.78)	
Futime, (25%-75%)	558 (351-1457)	612 (408-1145)	581 (364-1323)	0.393
**Fustat, n (%)**				0.242
alive	98 (66.22)	54 (73.97)	152 (68.78)	
dead	50 (33.78)	19 (26.03)	69 (31.22)	
**Grade, n (%)**				0.377
G1	16 (10.81)	11 (15.07)	27 (12.22)	
G2	70 (47.30)	26 (35.62)	96 (43.44)	
G3	55 (37.16)	33 (45.21)	88 (39.82)	
G4	7 (4.73)	3 (4.11)	10 (4.52)	
**Stage, n (%)**				0.456
Stage I	70 (47.30)	38 (52.05)	108 (48.87)	
Stage II	36 (24.32)	10 (13.70)	46 (20.81)	
Stage III	40 (27.03)	24 (32.88)	64 (28.96)	
Stage IV	2 (1.35)	1 (1.37)	3 (1.36)	
**T, n (%)**				0.401
T1	71 (47.97)	38 (52.05)	109 (49.32)	
T2	37 (25.00)	10 (13.70)	47 (21.27)	
T3	32 (21.62)	23 (31.51)	55 (24.89)	
T4	8 (5.41)	2 (2.74)	10 (4.52)	
**M, n (%)**				0.991
M0	146 (98.65)	72 (98.63)	218 (98.64)	
M1	2 (1.35)	1 (1.37)	3 (1.36)	
**N, n (%)**				0.991
N0	146 (98.65)	72 (98.63)	218 (98.64)	
N1	2 (1.35)	1 (1.37)	3 (1.36)	

Abbreviations: T: Tumor; N: Node (regional lymph node); M: Metastasis.

P-value > 0.05 indicates that there is no statistical difference in clinical data between training group and testing group.

### Identification of differentially expressed metabolic genes in the training set

**Figure 1:**
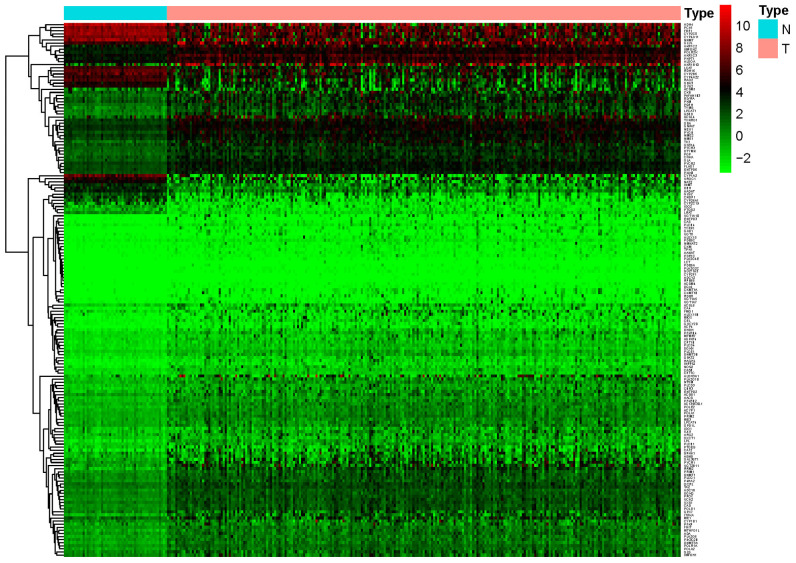
**Heat map of differentially expressed genes in the training set.** A total of 147 upregulated and 26 downregulated metabolic genes were identified in the tumor dataset compared to the normal dataset, using | logFC |> 1.5, *P-*value < 0.05, FDR < 0.05 as the screening criteria.

A total of 944 metabolic genes were extracted from the training set. After screening, we identified 173 differentially expressed metabolic genes in the tumor dataset, compared with the normal dataset, including 147 upregulated and 26 downregulated metabolic genes, using | logFC | > 1.5, *P-*value < 0.05, FDR < 0.05 as the screening criteria. The top 10 differentially expressed metabolic genes are illustrated in Table 2. A heat map representing these differentially expressed metabolic genes is shown in Figure 1.

**Table 2:** The top 10 up-regulated and down-regulated genes.

**Gene**	**ConMean**	**TreatMean**	**LogFC**	***P*-Value**	**FDR**
**Down-regulated genes**					
CYP26A1	6.5695	0.655653	-3.32478	1.44E-20	1.14E-19
CNDP1	6.560749	0.894874	-2.8741	4.44E-22	4.50E-21
CYP2C19	3.085339	0.430674	-2.84076	1.36E-15	5.00E-15
CYP1A2	162.061	24.16823	-2.74535	3.90E-23	5.74E-22
DBH	10.7885	1.70276	-2.66355	1.51E-23	2.50E-22
NAT2	25.52141	4.919365	-2.37516	1.94E-24	4.92E-23
LRAT	1.0943	0.223422	-2.29217	5.65E-24	1.21E-22
CYP2C8	449.4618	92.74891	-2.2768	1.96E-25	9.03E-24
AADAT	11.03725	2.482022	-2.15279	5.26E-24	1.15E-22
LCAT	68.11541	16.46416	-2.04865	4.16E-25	1.53E-23
**Up-regulated genes**					
CEL	0.033783	0.597751	4.145164	6.33E-15	2.15E-14
TYRP1	0.0086	0.1793	4.381821	2.01E-04	2.79E-04
CKMT1A	0.006888	0.150314	4.447752	6.00E-07	1.03E-06
MIOX	0.025308	0.650612	4.684158	8.17E-11	1.92E-10
ACP4	0.013881	0.386036	4.797518	7.10E-12	1.87E-11
CKMT1B	0.007145	0.224978	4.976801	8.85E-04	1.17E-03
GAD1	0.002933	0.122091	5.379313	4.09E-14	1.32E-13
ALDH3A1	0.752937	44.25674	5.877225	3.85E-04	5.21E-04
UGT1A10	0.00825	0.673831	6.351786	1.26E-03	1.64E-03
RDH8	0.002213	0.222531	6.652179	3.41E-07	5.94E-07

### Identification of prognosis-associated metabolic genes

**Figure 2:**
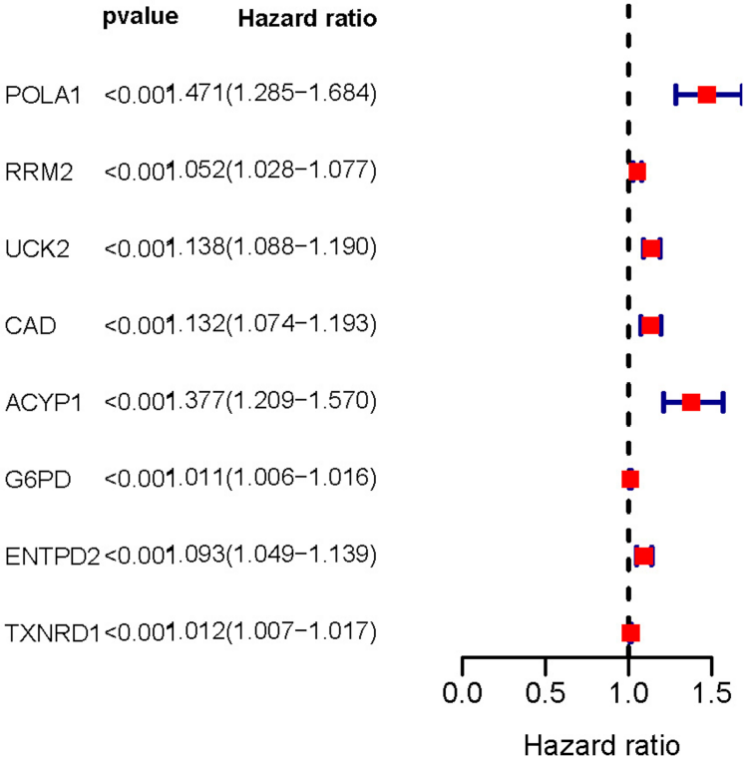
**Forest plot of prognosis-related metabolic genes screened using univariate Cox regression analysis.** The hazard ratio of these genes was > 1 indicating poor overall survival outcomes. *P-*value < 0.001.

In order to identify genes most closely related to prognosis in HCC, univariate Cox regression analysis was performed with the *P*-value strictly set to 0.001. A total of 8 prognosis-associated metabolic genes were identified in the training set, including *POLA1, RRM2, UCK2, CAD, ACYP1, G6PD, ENTPD2* and *TXNRD1.* The hazard ratio (HR) of these genes were > 1, which indicated that these genes were associated with higher risk of poor overall survival outcomes (Table 3 and Figure 2).

**Table 3:** Prognostic associated metabolic genes.

**ID**	**HR**	**HR.95L**	**HR.95H**	***P-*value**
POLA1	1.470951	1.284508	1.684457	2.40E-08
RRM2	1.052166	1.027998	1.076901	1.79E-05
UCK2	1.137793	1.088132	1.189719	1.43E-08
CAD	1.132149	1.074487	1.192906	3.26E-06
ACYP1	1.37746	1.208838	1.569603	1.53E-06
G6PD	1.010954	1.006035	1.015896	1.20E-05
ENTPD2	1.092836	1.048756	1.13877	2.38E-05
TXNRD1	1.012026	1.006645	1.017436	1.11E-05

### Construction of LASSO Cox regression model

A total of five prognosis-associated metabolic genes were enrolled in the LASSO Cox regression model, including *POLA1, UCK2, ACYP1, ENTPD2* and *TXNRD1*. The risk score = 0.224526000042292 *expression of *POLA1* + 0.0430802421098465 *expression of *UCK2 *+ 0.133617317495605 *expression of *ACYP1* + 0.0735585351730793 *expression of *ENTPD2* + 0.00710372438454236 *expression of *TXNRD1*. Patients were divided into high and low-risk groups based on the median risk score of the training set.

### Validation of training set survival analysis by utilizing testing set data

**Figure 3:**
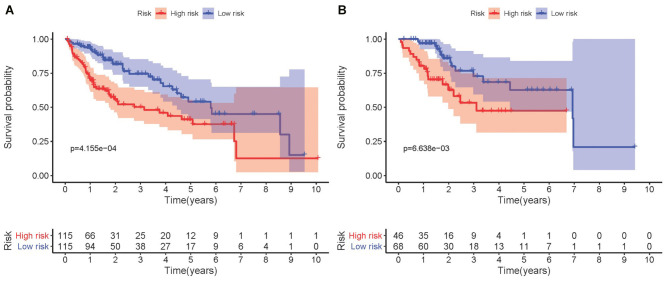
**Kaplan-Meier survival analysis involving prognostic metabolic genes in HCC.** (**A**) Kaplan-Meier curve of the training set. (**B**) Kaplan-Meier curve of the testing set. *P-*values were < 0.05, indicating that overall survival was significantly different between patients at high- and low-risk.

Kaplan-Meier analysis demonstrated that high-risk group members had a worse overall survival outcome compared to the low-risk group, in both the training (Figure 3A) and testing sets (Figure 3B). The *P-*values were < 0.05, which validated the effectiveness of the risk score in survival analysis based on the LASSO Cox regression model. Additionally, data from the Gene Expression Omnibus (GEO) database showed that the high-risk group had a poorer overall survival outcome (Supplementary Figure 1).

### Validation of risk score, survival status distribution, and heat map of the training set utilizing testing set data

**Figure 4:**
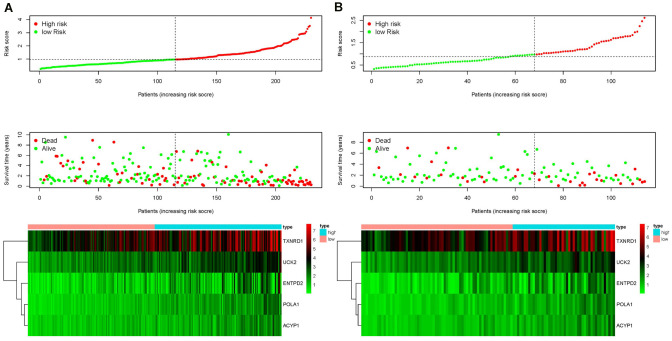
Ranking of risk scores, survival status distribution, and heat map for prognosis-associated metabolic genes of patients with HCC in the training set (**A**) and testing set (**B**), which demonstrated that the higher the risk score, the shorter the survival time and fewer the surviving patients, and the expression of the five prognostic genes was upregulated in high-risk groups in both the training and testing sets.

Each patient was ranked based on their risk score (top of Figure 4A, 4B). The risk score was elevated from left to right in both the training and testing sets. The distribution of the survival status of each patient demonstrated that the higher the risk score, the shorter the survival time and the fewer surviving patients in both the training and testing sets (middle of Figure 4A, 4B). Heat maps showed that the expression of the five prognostic genes was upregulated in high-risk groups in both the training and testing sets (bottom of Figure 4A, 4B). Validation of risk score, survival status distribution, and heat map generation using TCGA data were also performed by utilizing data from the GEO database. The result further confirmed the prognostic value of the risk score (Supplementary Figure 2).

### Univariate and multivariate Cox analysis

**Figure 5:**
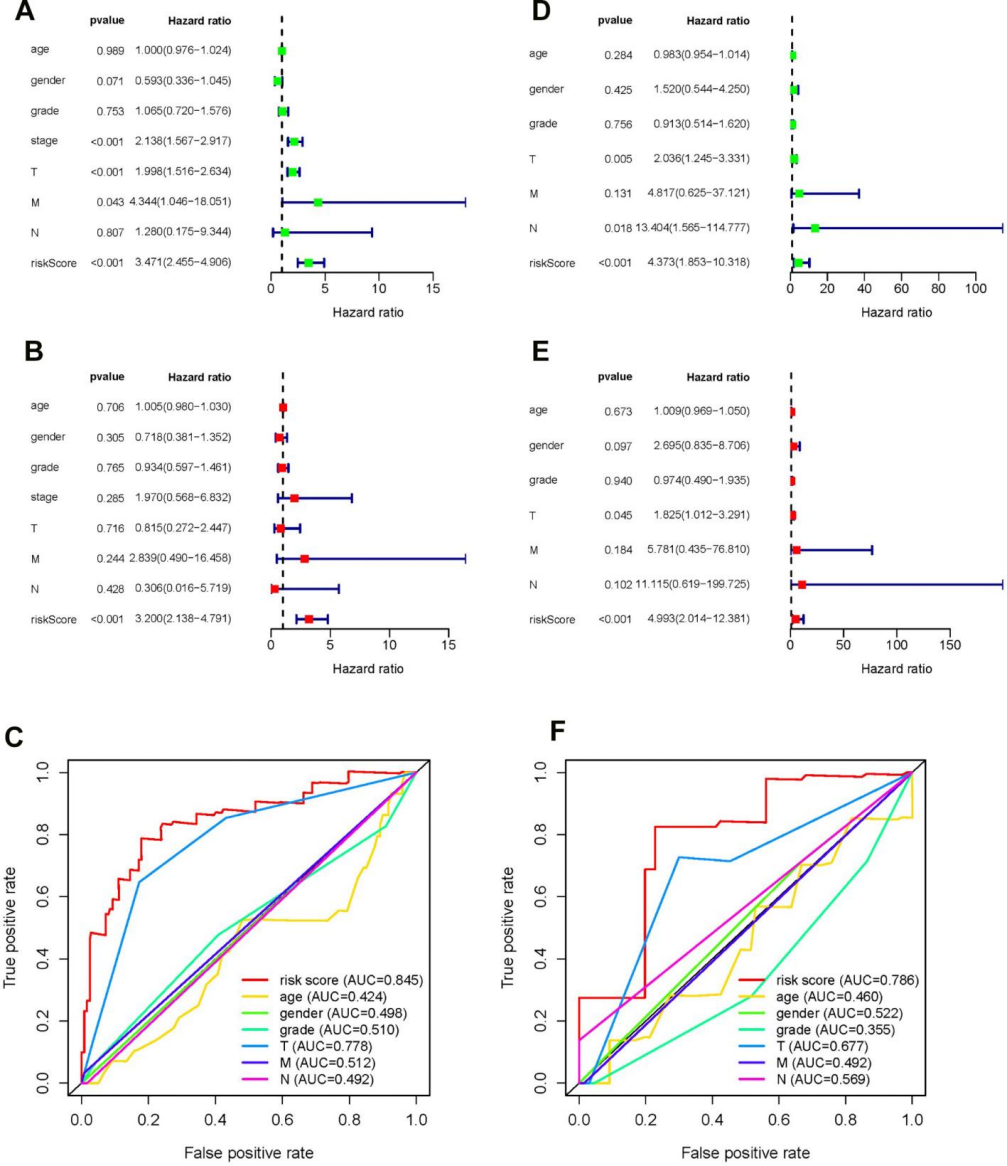
**Forrest plot of univariate and multivariate Cox regression analyses, and evaluation of receiver operating curve (ROC) analysis in HCC.** Forrest plot of univariate Cox regression analysis of the training set (**A**) and training set (**D**). Forrest plot of multivariate Cox regression analysis of the training set (**B**) and testing set (**E**). ROC analysis of the training set (**C**) and training set (**F**).

Univariate and multivariate Cox regression analyses were conducted to test whether the prognostic ability of the five metabolic genes was independent of clinical data in both the training and testing sets. For each dataset, we included age, gender, tumor grade, tumor stage, TNM staging, and risk score as explanatory variables. Univariate Cox regression analysis demonstrated that the risk score was related to the poor overall survival of patients with HCC (HR _training set_= 3.471, HR _testing set_ = 4.373, *P*-value < 0.05). As the risk score increased, the risk of poor survival outcomes elevated (Figure 5A, 5D). The results of multivariate Cox regression analysis showed that the risk score could be treated as an independent risk factor to predict overall survival outcome in patients with HCC (HR _training set_= 3.200, HR _testing set_ = 4.993, *P*-value < 0.05, Figure 5B, 5E). Receiver operating characteristic (ROC) analysis was performed to evaluate the efficacy of the multivariate Cox regression analysis. The area under the curve (AUC) of the risk score from the ROC curve was higher compared to other clinical characteristics (AUC _training set_ = 0.845, AUC _testing set_ = 0.786), implying a robust prognostic capacity of the constructed predictive model in predicting overall survival (Figure 5C, 5E).

### Gene Ontology (GO) and Kyoto Encyclopedia of Genes and Genomes (KEGG) analyses of the five metabolic genes

To determine the biological implications of the five metabolic genes, KEGG pathway and GO term analyses were performed. KEGG pathway analysis results showed that these five genes were associated with DNA replication and pyruvate metabolism. GO term enrichment indicated that the five genes were associated with nucleobase metabolic process such as pyrimidine nucleobase metabolic processes, DNA strand elongation, nucleoside kinase activity, and DNA polymerase activity (Table 4). Enrichment analyses demonstrated that the five metabolic genes may affect the prognosis of patients with HCC by inducing tumor cell proliferation.

**Table 4:** GO and KEGG analyses of the five metabolic genes.

	**Term**	**P-value**
**KEGG Pathway**	DNA replication	8.97E-03
	Pyruvate metabolism	9.71E-03
**GO-BP**	nucleobase metabolic process	1.50E-03
	pyrimidine nucleobase metabolic process	2.00E-03
	lagging strand elongation	2.00E-03
	pyrimidine nucleoside salvage	3.00E-03
	pyrimidine-containing compound salvage	3.00E-03
	DNA strand elongation	3.25E-03
	nucleoside salvage	3.50E-03
	pyrimidine nucleoside biosynthetic process	3.74E-03
	pyrimidine-containing compound metabolic process	3.74E-03
	nucleobase-containing small molecule catabolic process	3.74E-03
**GO-MF**	nucleoside kinase activity	3.50E-03
	DNA-directed DNA polymerase activity	4.99E-03
	DNA polymerase activity	5.24E-03
	nucleobase-containing compound kinase activity	7.48E-03

### External validation using online database

**Figure 6:**
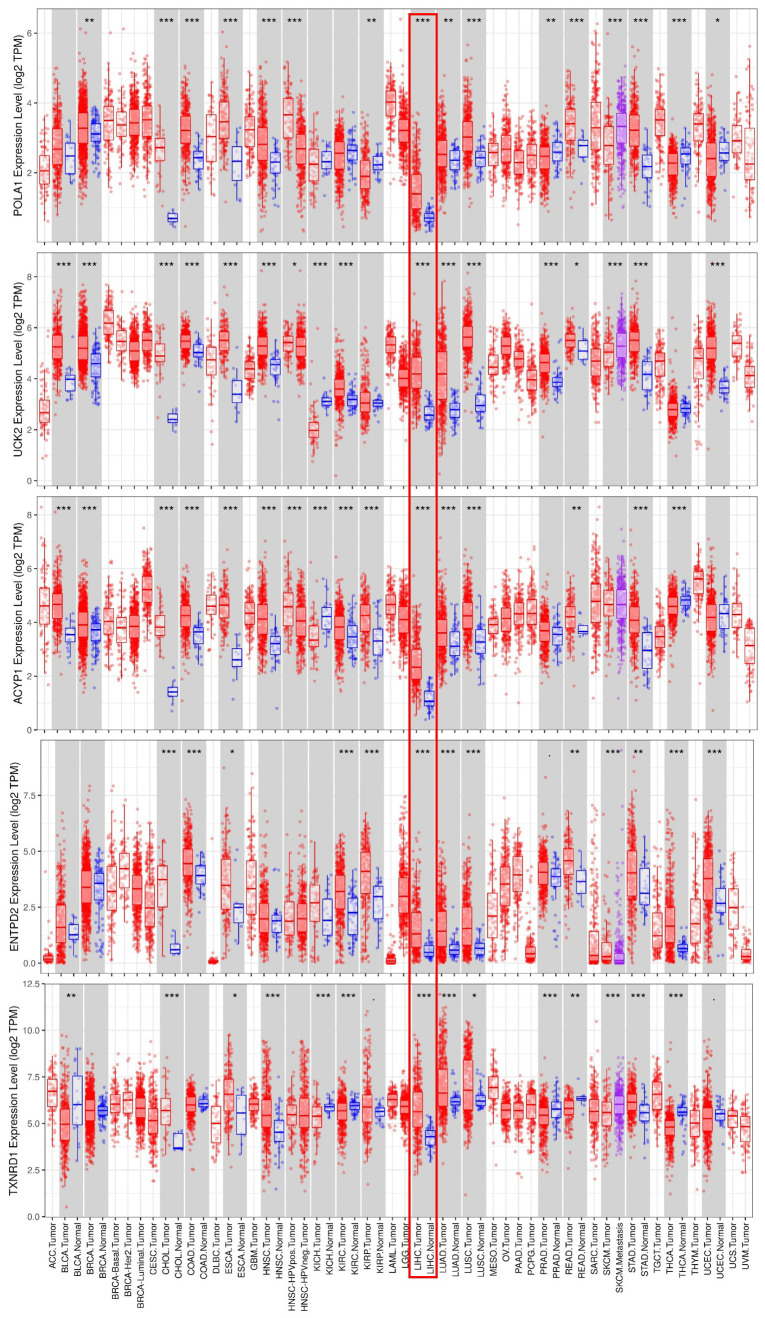
**mRNA expression of the five prognosis-related metabolic genes in cancers.** Data are from the TIMER database (https://cistrome.shinyapps.io/timer/) [50]. The expression of *POLA1, UCK2, ACYP1, ENTPD2 and TXNRD1* was significantly upregulated at the mRNA level in HCC.

**Figure 7:**
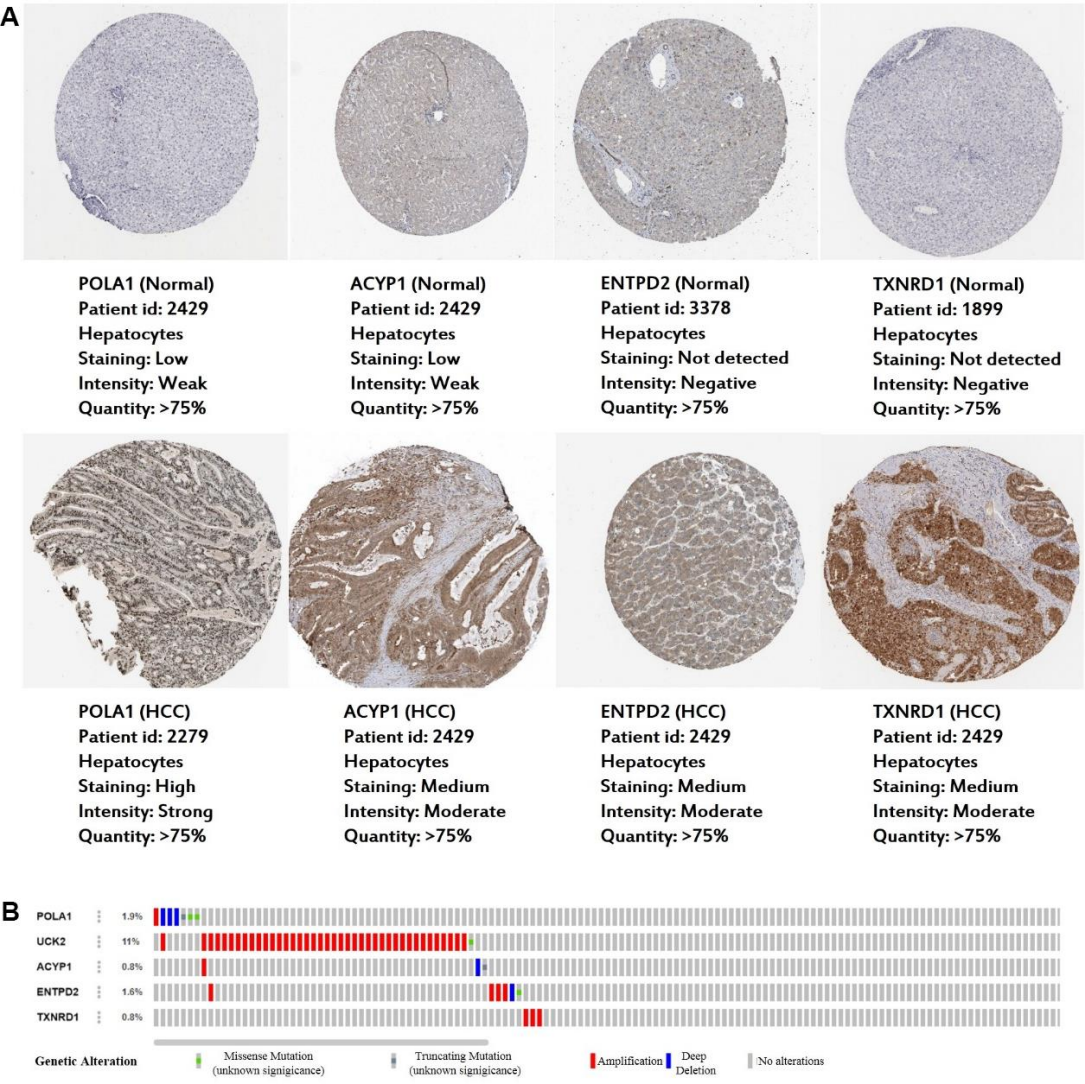
**Expression and genetic alterations in the five prognosis-related metabolic genes.** (**A**) The representative protein expression of the four genes in HCC and normal liver tissues. Data are from the Human Protein Atlas (http://www.proteinatlas.org) [51] database. Data for UCK2 were not found in the database. (**B**) Genetic alterations to the five genes in TCGA-LIHC. Data are from the cBioportal for Cancer Genomics (http://www.cbioportal.org/) [52, 53].

Consistent with our results, *POLA1, UCK2, ACYP1, ENTPD2* and *TXNRD1* were found to be significantly overexpressed at the mRNA level in HCC in the TIMER database (Figure 6). Protein expression, as evaluated using immunohistochemical staining, was explored using the Human Protein Atlas (HPA), which showed that POLA1, ACYP1, ENTPD2 and TXNRD1 were overexpressed in HCC (Figure 7A). UCK2 protein expression was not identified in HPA. However, genetic alterations curated in the cBioportal database demonstrate that *UCK2* possesses the most frequent amplification mutation in HCC (Figure 7B). Taken together, aberrant expression of the five genes in HCC was further validated.

### GO and KEGG analyses of high-risk patient genes using GSEA

**Figure 8:**
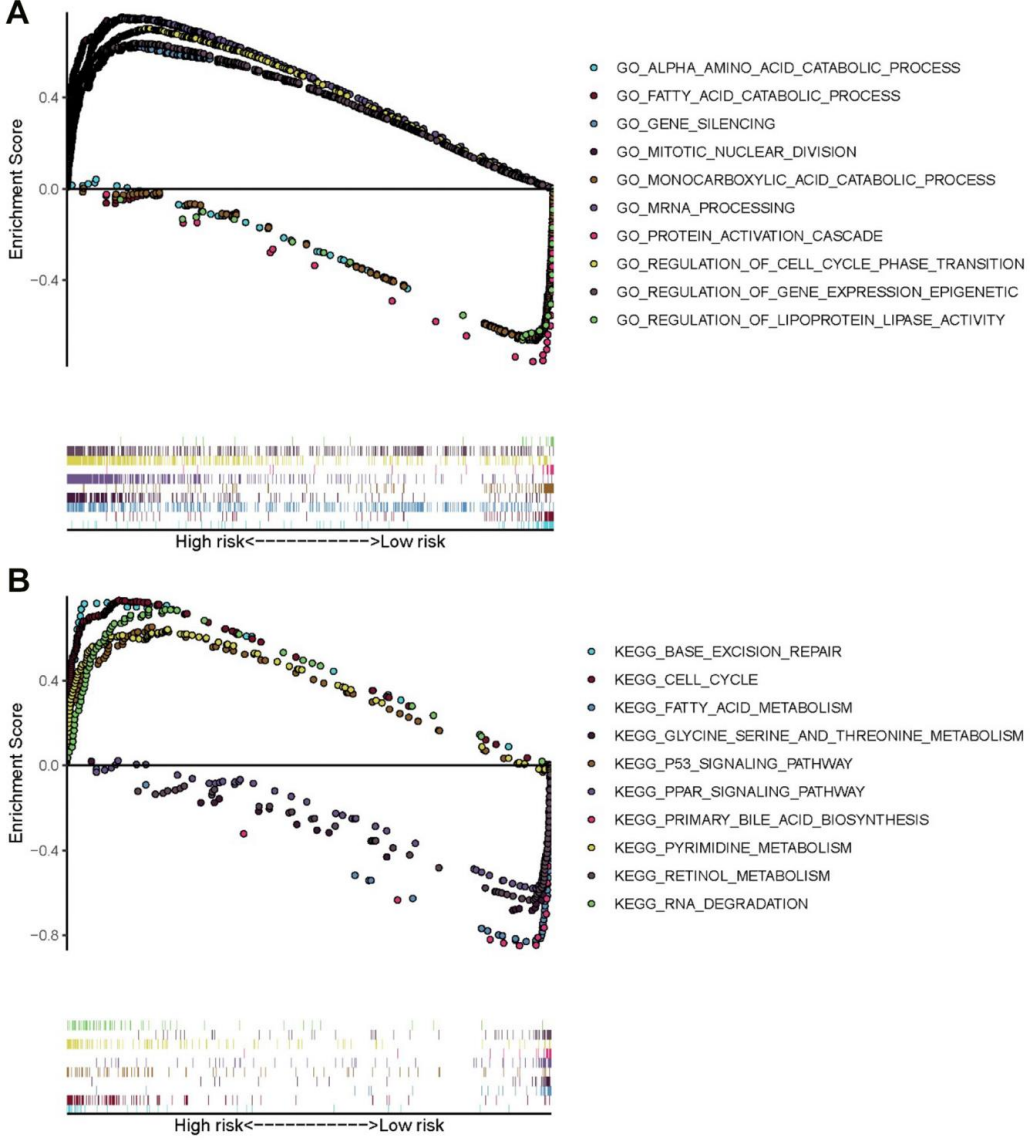
**GO and KEGG enrichment analyses via GSEA of the training set.** (**A**) Top five GO terms in high- and low-risk patients. (**B**) Five representative metabolism-associated KEGG pathways in high- and low-risk patients. The curves above the abscissa represent GO terms and KEGG pathways enriched by genes in high-risk patients.

To explore the potential mechanism of HCC pathological process, GO and KEGG analyses via GSEA were performed using the training set. The results of GO analysis demonstrated that genes of high-risk patients were mainly enriched in gene silencing, regulation of gene expression epigenetic, regulation of cell cycle phase transition, mitotic nuclear division, and mRNA processing, whereas genes of low-risk patients were mainly enriched in monocarboxylic acid catabolic process, protein activation cascade, fatty acid catabolic process, alpha amino acid catabolic process, and regulation of lipoprotein lipase activity (Figure 8A). The results of KEGG analysis showed that genes of high-risk patients were mainly enriched in cell cycle, p53 signaling pathway, RNA degradation, pyrimidine metabolism, and base excision repair. Genes of low-risk patients were mainly enriched in fatty acid metabolism, primary bile acid biosynthesis, peroxisome proliferator-activated receptor signaling pathway, retinol metabolism, and glycine, serine, and threonine metabolism. (Figure 8B).

### Development of a nomogram

**Figure 9:**
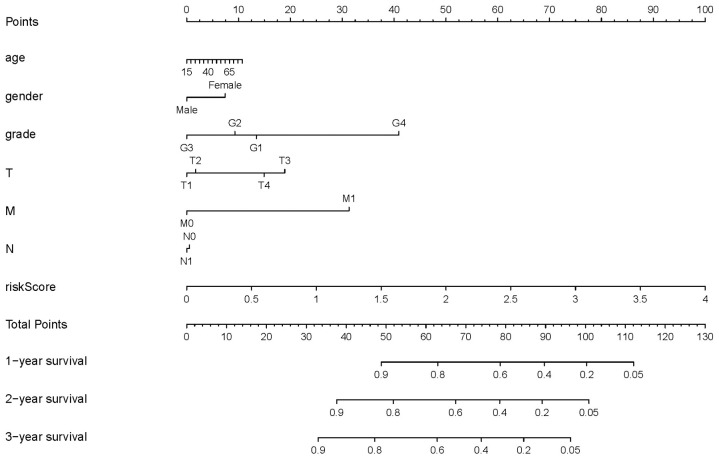
**Nomogram for predicting the survival of patients with HCC.** A straight line was drawn up to the axis labeled Points to determine the corresponding points. This process was repeated for each of the remaining axes, drawing a straight line each time to the Points axis. The points received for each predictive variable were added and this number was located on the Total Points axis. A straight line was drawn down from the Total Points to the 1-, 2- and 3-year survival axes to determine the predicted survival probabilities of patients.

Based on these results, we developed a predictive model, and generated a graphical nomogram with the training set data. Risk scores, combined with other clinical information, including age, gender, tumor grade, TNM staging, were incorporated into the nomogram to predict the probability of 1-, 2- and 3-year survival of patients with HCC (Figure 9). The C-index was 0.70, 0.77, and 0.83 for the T stage, risk score, and nomogram, respectively. The AUC of the nomogram for 1-, 2-, and 3-year OS were 0.91, 0.88 and 0.89, respectively (Table 5). This indicated that combining our prognostic signature with TNM staging increased the AUC for predicting 1-, 2-, and 3-year OS.

**Table 5:** Comparison of the nomogram with the TNM and risk score.

**Model**	**T**	**N**	**M**	**risk score**	**Nomogram**
1-year AUC	0.77	0.49	0.51	0.80	0.91
2-year AUC	0.71	0.49	0.51	0.78	0.88
3-year AUC	0.75	0.49	0.52	0.75	0.89
C-index	0.70	0.51	0.50	0.77	0.83

## DISCUSSION

### Metabolic deregulation as an emerging cancer cell hallmark

HCC is the most common primary liver cancer, and the third leading cause of cancer deaths worldwide [14]. Due to limited symptoms in the early stages, and limitation of current biomarkers, 75% of patients with HCC are usually diagnosed at an advanced stage, with a pessimistic overall survival rate [15]. Additionally, the treatment responses of patients in advanced stages are usually poor [16]. Hence, it is essential to diagnose HCC at the early stage, and there is an urgent need to develop novel diagnostic or prognostic biomarkers for HCC. Compelling evidence has suggested that metabolic deregulation is an emerging hallmark of cancer cells because of its important roles in cell growth, proliferation, angiogenesis, and invasion [17]. Accumulating evidence demonstrates that the metabolic alterations in neoplastic cells are closely related to mortality risk in cancer [18, 19]. Based on this, we screened five novel prognosis-related metabolic genes, and constructed an overall survival predictive model using data of patients with HCC from TCGA database. Univariate and multivariate Cox regression analyses suggested that the prognostic capacity of the five metabolic genes was independent of other clinical data. Kaplan-Meier analysis and the AUC in the ROC curve demonstrated effective stratification of low- and high-risk patients according to different overall survival results, suggesting robust prognostic value involving these metabolism-related genes.

### Genetic testing of prognosis-related genes is more accurate, convenient, and affordable for cancer diagnosis and treatment

Recent studies have shown that clinical features such as age, gender, tumor grade, and metastatic diagnosis are insufficient to accurately predict the outcome of patients with HCC [20]. TNM staging is still an important clinical method for predicting the prognosis of patients with HCC. In our analysis, the AUC of risk score was larger than TNM staging in both the training and testing sets. Furthermore, we developed an easy-to-use nomogram integrating risk score and other clinical information to facilitate the prediction of overall survival. In the current study, the combined nomogram for predicting 1-, 2-, and 3-year OS was superior to both the gene signature and TNM staging, with a higher C-index and AUC of ROC. Moreover, we further compared our prognostic gene signature with other gene signatures. The AUC of a Long et al. (2018) [21] model is 0. 7674, 0.7040, 0.6919 at 1, 3 and 5-years, and the AUC reported in a model by Qiao et al. (2019) [22] is 0.71, 0.69 at 3 and 5-years. ROC analysis indicated that our model had better performance, where the AUC for 1-, 2-, 3-years was 0.8, 0.78 and 0.75, respectively. The prognostic model in this study substantially improved the accuracy of HCC diagnosis, thereby providing a reliable basis for formulating a reasonable treatment plan. Recently, gene signatures based on aberrant mRNA have gained much attention and shown great potential in cancer prognosis [23, 24]. Gene combination testing can complete the genetic analysis of patients, and facilitate treatment planning accordingly, which has promoted the realization of individualized treatment [25]. Additionally, as could be easily acquired using mRNA of merely five genes, such signatures could be a cost-effective complement to expensive metabolic imaging such as (18)F-fluorodeoxyglucose-positron emission tomography to reflect metabolic activity in HCC [26, 27]. Therefore, carrying out genetic testing can be more advantageous, providing guidance to improve cancer treatment efficiency.

### Prognosis-related metabolic genes, and GO and KEGG enrichment analyses lay down the groundwork for future therapeutic approaches

GSEA results demonstrated that the functions and pathways of upregulated genes of patients at a high-risk of HCC were mainly focused on the regulation of nucleic acid metabolism, as well as fatty acid metabolism. A total of five prognosis-associated metabolic genes were included in the LASSO Cox regression model, including *POLA1, UCK2, ACYP1, ENTPD2* and *TXNRD1*. Furthermore, functions and pathways related to these genes echoed the results of GSEA: Nucleotide metabolism and *POLA1, UCK2, ENTPD2* and *TXNRD1*: Unrestricted cell proliferation is a characteristic typical of cancer. Purine and pyrimidine are the basic components of nucleotides in cell proliferation; therefore, impaired purine and pyrimidine metabolism is associated with the cancer progression [28]. *POLA1* encodes DNA polymerase α, the enzyme responsible for initiating DNA synthesis during the S phase of the cell cycle [29]. The encoded protein of *UCK2* catalyzes phosphorylation of uridine and cytidine to uridine monophosphate (UMP) and cytidine monophosphate (CMP), respectively [30]. Ectonucleoside triphosphate diphosphohydrolase 2 (*ENTPD2*) is related to pathways involved in ATP/ITP metabolism, and metabolism of nucleotides [31]. Pathway that related to *TXNRD1* is gene expression [32]. Many researchers have shown that these genes promote HCC cell migration and invasion, are associated with poor patient survival, and might represent novel potential targets in HCC therapy [33–42]. Fatty acid metabolism and *ACYP1*: Cancer cells must rewire cellular metabolism to satisfy the demands of growth and proliferation. In addition to the indefinite proliferation of tumor cells, the expression of metabolic enzymes may also be regulated by increases in gene copy number in cancer cells [43]. The overexpression of *ACYP1,* which is involved in lipid metabolism, is associated with unfavorable prognoses in patients with HCC [44]. With the development of gene therapy, it is also a beneficial to adopt genetic inhibition of these metabolic genes to prevent the proliferation of HCC cells and induce apoptosis *in vitro*.

External information from online databases validated that the expression of these five prognosis-related metabolic genes is upregulated at the DNA, mRNA and protein levels, which is consistent with our analyses. In future, further experiments will be performed to explore the roles of these five metabolic genes as a whole in the pathogenesis, prognosis, and treatment of HCC.

## CONCLUSIONS

Taken together, our results have identified five prognosis-related metabolic genes useful for predicting survival outcomes in HCC, based on TCGA data, which reflected that these genes might be involved in dysregulation of the metabolic microenvironment, and might be treated as novel biomarkers for metabolic therapy in HCC patients.

## MATERIALS AND METHODS

### Downloading mRNA expression profiles and clinical information

RNA sequencing data of HTSeq-FPKM and relevant clinical information of our HCC cohort were downloaded from TCGA database (http://portal.gdc.cancer.gov/). Next, the entire TCGA-HCC data set was randomly divided into a training set and a testing set (Table 1), both of which had similar clinical characteristics.

### Extraction of metabolic genes from the TCGA-HCC database

The file c2.cp.kegg.v7.1.symbols.gmt was downloaded from the GSEA website (http://software.broadinstitute.org/gsea/index.jsp). The metabolic genes were obtained from METABOLISM pathways in c2.cp.kegg.v7.1.symbols.gmt. Then the mRNA expression of metabolic genes in the TCGA-HCC database was extracted.

### Identification of differentially expressed metabolic genes in the training set

The R package “limma” was used to screen the differentially expressed metabolic genes [45]. The expression of candidate metabolic genes in the training set was used to identify differentially expressed metabolic genes. The screening criteria was set as |logFC| > 1.5, *P-*value < 0.05 and FDR < 0.05.

### Identification of prognostic associated metabolic genes in the training set

Univariate Cox regression analysis was performed using the R package "survival" to determine metabolic genes related to prognosis in the training set. The overall survival outcomes of genes with hazard ratio (HR) <1 are better, while the overall survival outcomes of genes with HR> 1 are worse. The statistical significance was based on *P-*value < 0.001.

### Construction of Lasso Cox regression model

The R package “glmnet” and “survival” were used for the construction of Lasso Cox regression model. To calculate the risk score of every patient, Lasso Cox regression model was constructed with the prognostic related metabolic genes screened by univariate Cox regression analysis [46]. The formula of risk score was as follows: *risk score* = the sum of each coefficient of mRNA multiple each expression of mRNA. Patients were divided into high and low-risk groups based on the median risk score of the training set.

### Survival analysis based on the stratification of low and high-risk scores

The Kaplan-Meier method was used for survival analysis, and log-rank test was used to evaluate the overall survival difference between high and low-risk groups. The R package “survival” and “survminer” were used to perform survival analysis. Risk score curves were generated according to the risk score of each patient. In order to validate the survival analysis of the training set, the Kaplan-Meier method was performed on the testing set to evaluate the overall survival difference between high and low-risk groups.

### Validation of risk score via univariate and multivariate Cox analysis

Clinicopathological characteristics and risk scores were included in univariate and multivariate Cox regression analyses to validate whether the risk score could be regarded as an independent risk factor to predict overall survival outcome. This factor can be used as independent risk factor when *P-*values < 0.05.

### Validation of risk score by drawing receiver operating characteristic curve

R package “survivalROC” was used to draw receiver operating characteristic curve (ROC curve). The robustness of risk score for overall survival prediction model was evaluated by comparing the area under the curve (AUC) in the ROC curves of clinicopathological characteristics and risk score.

### GO analysis

GO analysis can be divided into three parts: molecular function, biological process, and cellular component, which respectively describe the molecular functions of potential gene products, the biological processes involved, and the cellular environment in which they are located. Enrichment analysis was performed via David v.6.8 (https://david.ncifcrf.gov/) database [47, 48]. David v.6.8 for annotation, visualization, and integrated discovery provides a comprehensive set of functional annotation tools to understand the biological meaning behind long lists of genes.

### Pathway analysis

The Kyoto Encyclopedia of Genes and Genomes (KEGG) enrichment analysis of prognostic genes can enrich the significant pathways and help to find the biological regulatory pathways for significant differences in experimental conditions. The David 6.8 (https://david.ncifcrf.gov/) database also can be used for the enrichment of pathway.

### External validation of the prognostic genes expression using online database

The expression of the prognostic genes in the gene signature was further validated at the DNA level in the cBioportal database (http://www.cbioportal.org/), at the mRNA level in the TIMER database (https://cistrome.shinyapps.io/timer/), and at the protein level in the Human Protein Atlas database (https://www.proteinatlas.org/).

### GO and KEGG analyses by GSEA

GSEA v4.0.3 for Windows, c5.bp.v7.1.symbols.gmt and c2.cp.kegg.v7.1.symbols.gmt were downloaded from the GSEA website (http://software.broadinstitute.org/gsea/index.jsp). GSEA software (version 4.0.3) was used to perform GO and KEGG analyses [49]. The gene sets databases c5.bp.v7.1.symbols.gmt and c2.cp.kegg.v7.1.symbols.gmt were selected as GO and KEGG GMT files, respectively. CLS file was prepared according to the high and low-risk groups based on the median risk score of training set. GCT file was prepared with the mRNA expression matrix of HCC downloaded from the TCGA database. And permutations was set as 1000.

### Development of risk prediction model

According to training set data, we developed a nomogram combing risk scores with clinical information for prediction of overall survival at 1, 2 and 3 years for patients with HCC. The R package “rms” was used to produce the nomogram. The concordance index (C-index) were used to investigate the discrimination of the nomogram (by a bootstrap method with 1,000 resamples). Tumor-node metastasis (TNM) staging, risk score, and the combined model including TNM and the risk score were compared with time-dependent receiver operating characteristic curve (time-ROC curve), C-index.

### Statistical methods

Independent sample *t*-test and nonparametric independent sample test were performed on the clinical data of the training set and testing set, to evaluate statistical difference of clinical data between the two groups. Survival analysis was performed using Kaplan-Meier method, and log-rank test was used to evaluate the overall survival difference between high and low-risk groups. Univariate and multivariate Cox regression analyses were implemented to examine whether the prognostic value of metabolic genes signature was independent. To assess the prognostic performance of the metabolic genes risk score signature, we conducted receiver operating characteristic (ROC) analyses.

## Supplementary Material

Supplementary Materials

Supplementary Figures
