# Supplementary material for: Prognostic roles of metabolic reprogramming-associated genes in patients with hepatocellular carcinoma
Source: Aging (Albany NY). 2020 Nov 12;12(21):22199–219. doi: 10.18632/aging.104122 (PMC7695384; doi:10.18632/aging.104122)
Supplement: Supplementary Materials [file aging-12-104122-s001..pdf]

SUPPLEMENTARY MATERIALS

METHODS

Downloading mRNA expression profiles and clinical information

We downloaded 387 cases from the GEO database and obtained the gene expression matrix of the GSE10143 series. The cases of non-hepatocellular carcinoma were deleted and there were 80 cases of hepatocellular carcinoma. The mRNA expression profiles and clinical information of the 80 cases were obtained.

RESULTS

Validation of training set survival analysis by utilizing data from the GEO database

Data from the GEO database (GSE10143) showed that high-risk group had a worse overall survival outcome by Kaplan-Meier analysis (Supplementary Figure 1). The *P*-values were < 0.05, which validated the effectiveness of the risk score in survival analysis based on the lasso Cox regression model.

Validation of risk score, survival status distribution and heatmap of the training set by utilizing data from the GEO database

Every patient was ranked based on the risk score (top of Supplementary Figure 2). The risk score was elevated from left to right. The distribution of the survival status of each patient demonstrated that the higher the risk score, the shorter the survival time and the fewer alive patients in GSE10143 dataset (middle of Supplementary Figure 2). The heatmaps showed that the expression of the five prognostic genes was up-regulated in high-risk groups in GSE10143 dataset (bottom of Supplementary Figure 2).
