# Supplementary material for: Prognostic roles of metabolic reprogramming-associated genes in patients with hepatocellular carcinoma
Source: Aging (Albany NY). 2020 Nov 12;12(21):22199–219. doi: 10.18632/aging.104122 (PMC7695384; doi:10.18632/aging.104122)
Supplement: Supplementary Figures [file aging-12-104122-s002..pdf]

SUPPLEMENTARY FIGURES

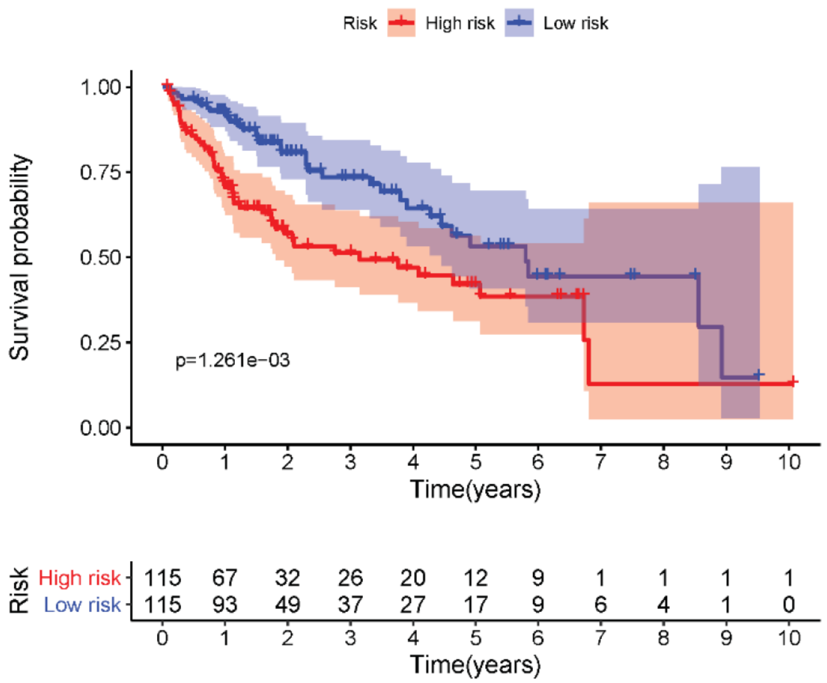

Supplementary Figure 1. Survival analysis by Kaplan-Meier method of the prognostic metabolic genes in HCC. The *P*-values were < 0.05, indicating the overall survival was significantly different between patients with high-risk and low-risk.

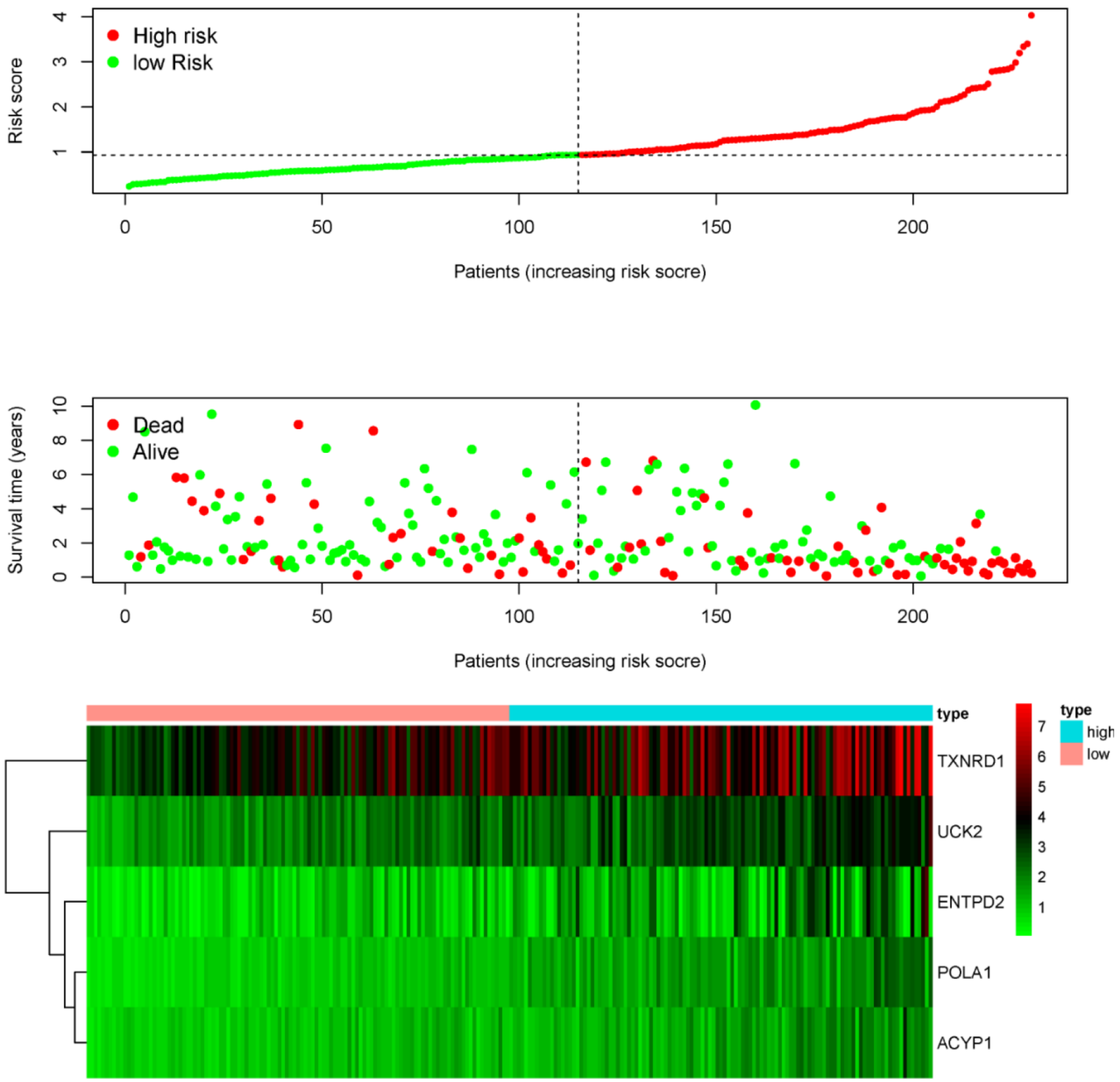

Supplementary Figure 2. The ranking of risk score, survival status distribution and heatmap for prognostic associated metabolic genes of patients with HCC in GSE10143 dataset, which demonstrated that the higher the risk score, the shorter the survival time and the fewer alive patients.
